# BRCA1 mutation- oncological treatment- reconstructive surgery of the breast- pregnancy: diagnostic and therapeutic procedures in a 28 year old patient diagnosed with a tumor in the left breast with a BRCA1 mutation

**DOI:** 10.1186/1897-4287-10-S1-A9

**Published:** 2012-01-12

**Authors:** Ewa Kilar

**Affiliations:** 1 Swidnica, Poland

## Process of diagnostic procedures (oncological approach)

Phase I: Primary diagnosis

1. Fine Needle Biopsy of tumor in left breast number 1666/2007- cellular neoplasm (september 2007).

2. Thick needle biopsy 4.10.2007- indeterminate results.

Phase II: Surgical diagnoses

1. Quadrant resection of left mammary gland with tumor (T2N0Mx) (October 12,2007) hisological pathology number 14946 Infiltrative ductal carcinoma (8 points).

Receptor results in neoplastic cells of the tumor in the left breast (October 12, 2007).

Receptors: Estrogen (+) Progesterone (-) Her2/Neu(-).

2. Lymphoscintography (October 25, 2007)- resection of sentinel lymph node (using gamma camera lymph node was located from the left axilla, where isotope was located).

Histopahological results of the sentinel lymph node showed reactive lymphadenitis.

Phase III: Diagnosis after surgery (genetic approach)

Opolskie Center of Oncology ul. Katowicka 66a oraz w MCND PUM w Szczecinie.

1. In family history BRCA 1 mutation in mother who was during treatment of invasive carcinoma of the mammary gland at that time.

2. Patients molecular results- BRCA1 mutation.

Phase IV: Diagnosis after genetic consultation

Gynecological exam, transvaginal ultrasound (11.12.07).

Ultrasound of breast (11.12.07)- left mammary gland, after surgical removal of tumor, neighboring fluid levels 0.4x3.27cm.

Marker CA-125- 11.5 U/ml (11.10.07).

MRI of breast (11.17.07)- neighboring with the surgical scar within left breast medially and anteriorly seen fluid 2x2x2.2cm.

Treatment of patient in a patient with BRCA1 mutation (oncological approach).

Qualified for Cis Platin chemotherapy (4 cycles of Cis Platin- from November to February 2008) before surgery.

Qualified for surgery (03.14.08) performed at the oncological center in Gdansk, profilactic subqutanous mastectomy of right breast, also subqutanous mastectomy of left breast (s.p. surgical removal of ductal ca.) simultanously with reconstruction of both breasts.

After recontructive surgery of breasts patient was under care of the Oncological Outpatient Clinic that is part of "Latawiec" hospital in Swidnica.

## Patient after treatment active in her career

Decision about pregnancy and medical consultation (gynecological approach).

1. October 2009 patient decided to become pregnant and 26 July 2010 had a healthy girl with Down Syndrome (kariotyp 47, XX,+21).

2. During pregnancy patient consulted a gynecologist but did not find a motive to terminate pregnancy.

3. Patient did not have amniocentesis. Program for amnicentesis qualifies patients from age 35.

4. Patient during pregnancy and currently active in her career.

## Discussion

New element of oncological treatment was to decide if the use Cis Platin before surgery was the right form of treatment.

Resulted in a esthetic effect after reconstructive surgery of breasts.

It was kept in mind that there could be negative effects during pregnancy (hormonal changes) and after pregnancy in patients gynecological health.

Patient was part of decision making throughout treatment and possibilities of side effects in patient and the child.

## Results

Use of Cis Platin as a first choice together with surgical treatment gives beneficial results in treatment of patient with BRCA1 mutation.

It is not clear in what degree the treatment affected the childs development during gestation.

Seems necessary that decides to get pregnant after oncological treatment should undergo amnicentesis, independent of patients age, to prevent possible teratogenic effects in the development of the fetus.

